# An optimized relational database for querying structural patterns in proteins

**DOI:** 10.1093/database/baad093

**Published:** 2024-01-17

**Authors:** Renzo Angles, Mauricio Arenas-Salinas, Roberto García, Ben Ingram

**Affiliations:** Department of Computer Science, Faculty of Engineering, Universidad de Talca, Camino a Los Niches Km. 1, Curicó, Región del Maule 3340000, Chile; Millennium Institute for Foundational Research on Data (IMFD), Vicuña Mackenna 4860, Macul, Santiago, Región Metropolitana 7810000, Chile; Centro de Bioinformática y Simulación Molecular (CBSM), Faculty of Engineering, Universidad de Talca, Av. Lircay s/n, Talca Región del Maule 34600000, Chile; Millennium Institute for Foundational Research on Data (IMFD), Vicuña Mackenna 4860, Macul, Santiago, Región Metropolitana 7810000, Chile; Engineering Systems Doctoral Program, Faculty of Engineering, Universidad de Talca, Camino a Los Niches Km 1, Curicó, Región del Maule 3340000, Chile; School of Water, Energy and Environment, Cranfield University, College Road, Cranfield, Bedfordshire MK43 0AL, England

## Abstract

A database is an essential component in almost any software system, and its creation involves more than just data modeling and schema design. It also includes query optimization and tuning. This paper focuses on a web system called GSP4PDB, which is used for searching structural patterns in proteins. The system utilizes a normalized relational database, which has proven to be inefficient even for simple queries. This article discusses the optimization of the GSP4PDB database by implementing two techniques: denormalization and indexing. The empirical evaluation described in the article shows that combining these techniques enhances the efficiency of the database when querying both real and artificial graph–based structural patterns.

## Introduction

A database is a crucial component in almost any software system. However, a functional database is not only about data modeling and schema design. It also involves database optimization or tuning, which means making a database application run faster. There are various methods available for optimizing databases, such as query rewriting, denormalization and indexing (33).

In the context of protein engineering and biotechnology, structural patterns are three-dimensional structures found in biological molecules, like proteins or nucleic acids. These patterns play a crucial role in comprehending the functionality of these molecules (13). The discovery and characterization of structural patterns is an important topic of research as it provides fundamental details about the functions of a protein and serves as a valuable tool for deciphering new proteins (27).

Additionally, there is the representation of interactions in protein–ligand patterns (38). Ligands are small molecules that interact with the amino acids of a protein, and they can interact, bind or control the biological functions of the protein. Identifying common patterns among different proteins allows for the discovery of new functions within them, as well as new methods for drug discovery (21, 26). This knowledge can then be applied in the treatment of diseases (9).

Numerous software solutions exist for identifying structural patterns in biological data, such as PyScoMotif (10), GeoMine (12) and Motif (7). Each of these tools has its unique interface and usability characteristics. For instance, PyScoMotif operates via a command-line interface, offering robust functionality for those comfortable with such an environment. On the other hand, options like GeoMine provide a graphical user interface, although they can be complex and less intuitive for some users. In response to these varying usability challenges, we developed a new software package, called GSP4PDB, that not only encompasses the core functionalities of these existing tools but also features a user-friendly interface. This design aims to make the complex task of pattern identification in structural biology more accessible and efficient for a broader range of users.

GSP4PDB (4) is a web application designed to simplify the search of protein–ligand structural patterns by utilizing a graph-based representation. In order to manage protein information, GSP4PDB employs a relational database hosted in PostgreSQL. This database contains data taken from the Protein Data Bank (PDB) (29), a database of proteins organized into files. These files pose a challenge when attempting to represent them in a relational database (34).

Various performance tests were conducted using different structural patterns in order to assess the performance of the relational database. These tests revealed that the system either could not provide a response or did so with long response times. As a result, we concluded that the normalized design employed in this implementation is inefficient and causes inadequate system response in certain scenarios, thus necessitating database tuning.

The goal of this study is to identify, describe, analyze and apply optimization techniques for relational databases in order to enhance the efficiency of query resolution in GSP4PDB. More specifically, two techniques were implemented. The first technique involved denormalizing the database tables, which means that redundant information was included in each table to minimize the time taken for queries. Second, indexes were added to the attributes of the tables that are frequently used during the search for structural patterns.

The empirical evidence from this work demonstrated that the optimization techniques employed effectively enhanced the overall efficiency of the system. Additionally, we provide evidence that a relational database system is capable of storing and querying intricate protein data presented in a graph format.

The remaining part of the article is organized as follows: Various concepts related to protein structural patterns are presented in the ‘Structural patterns in proteins’ section. The work related to this topic is presented in the ‘Related work’ section. A relational database for proteins, which has been normalized, is described in the ‘Relational database for proteins’ section. The optimization of the database is explained in the ‘Database optimization’ section. The experimental evaluation is presented in the ‘Experimental evaluation’ section. The conclusions and future work are discussed in the ‘Conclusions and future work’ section.

## Structural patterns in proteins

Proteins (24) are large and complex molecules that consist of a sequence of numerous units referred to as amino acids. Generally, proteins perform their biological functions by physically interacting with other molecules called ligands. ‘Computational Protein Design’ is a relatively new approach, which aims to merge physical chemistry models with state-of-the-art computational algorithms in order to automate the process of redesigning sequences of proteins (31). Some of the primary challenges in computational protein design include handling substantial amounts of biological data (14), as well as the high computational expenses associated with searching through various protein structures and patterns (15).

The notion of ‘structural pattern’ is employed to explain a three-dimensional structure or shape that appears in the secondary structure of a protein (8). The structure with the same pattern can be found in a group of proteins with a particular frequency and meeting specific criteria such as atomic distance, composition and connectivity.

### Protein–ligand structural patterns

A ‘protein–ligand structural pattern’ (37) is described as the union of a ligand and a group of amino acids. The arrangement of these components in three-dimensional space can be determined by three types of relationships: the distance between two amino acids, the distance between an amino acid and the ligand and the order in which amino acids appear in the sequence relative to other amino acids. As an example, a zinc finger (19) is a protein–ligand structural pattern in which a zinc atom serves as the ligand and is surrounded by cysteine (CYS) and histidine (HIS) amino acid residues.

**Figure 1.**
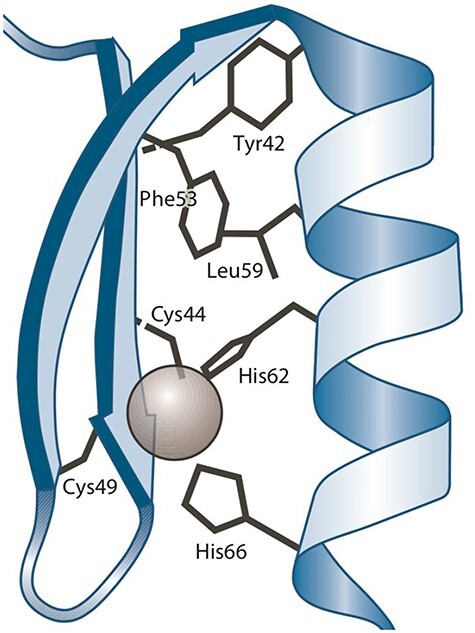
Three-dimensional representation of the Zinc finger pattern (20). Alt Text: A Zinc finger pattern.

Figure 1 shows a three-dimensional depiction of the zinc finger of C_2_H_2_, showcasing the tetrahedral coordination of zinc with two CYSs and two HISs.

Structural patterns are typically represented using text (i.e. a textual notation). One example is the PA (Pattern) line notation^1^ defined by PROSITE, which can be employed to represent the zinc finger pattern mentioned earlier as C-x(2,4)-C-x(12)-H-x(2,6)-H. Note that the graphical representation (Figure 1) is well suited to visualize the protein–ligand interaction (including some structural details); however, it is not useful to draw, modify and share.

Furthermore, the textual representation offers a straightforward syntax for describing the structure of the subsequence that takes part in the binding site. However, it is unable to describe certain aspects of the interaction, such as the distances between the ligand and amino acid.

Taking into account the problems outlined earlier, we will now introduce a graph-based model that can represent structural patterns of proteins and ligands.

### Graph-based structural pattern

**Figure 2.**
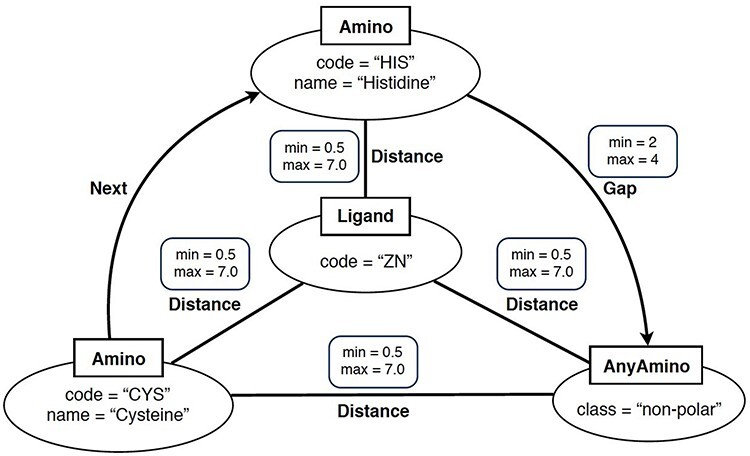
Example of graph-based structural patterns. It shows a zinc ligand connected with two specific amino acids (CYS and HIS) plus an undefined amino acid. Alt Text: A graph-based structural pattern.

In simple terms, a ‘graph-based structural pattern’ (GSP) is a graph in which the nodes depict the components of a protein (such as amino acids and ligands) and the edges represent the structural relationships (such as the distance between amino acids) (3). An example of GSP is illustrated in Figure 2.

Formally, a GSP is a property graph. This refers to a graph where nodes and edges can have properties represented as name–value pairs. A GSP permits four types of nodes: Amino nodes, AnyAmino nodes, Ligand nodes and AnyLigand nodes. Furthermore, GSP allows three types of edges: Distance edges, Next edges and Gap edges.

As shown in Figure 2, nodes are represented as ellipses whose label (inside a square) determines their type. An Amino node represents a specific amino acid, whose name is defined by the property name. An AnyAmino node represents the occurrence of an unfixed amino acid (as a wildcard). Each AnyAmino node includes the property polarity, whose value can be ‘any’, ‘non-polar’, ‘polar uncharged’, ‘positively charged’ or ‘negatively charged’. A Ligand node represents the ligand of the pattern, whose three-letter identifier is defined by the property code. An AnyLigand node represents an unfixed ligand (similar to an AnyAmino node).

Edges can be directed or undirected and are labeled with their type. A Distance edge is an undirected edge which represents the distance relationship between two amino acids or between an amino acid and the ligand. A Distance edge includes the properties min and max, which allow us to define the minimum and maximum distances, whose default values are 0.5 and 7 Angstroms (Å), respectively. A Next edge is a directed edge which allows us to specify that a node *X* follows a node *Y* in the protein chain (i.e. they are neighbors). A Gap edge is a directed edge which represents the occurrence of a given number of amino acids between two specific amino acids. The number of amino acids is defined by the properties min and max, satisfying that min > 0, max ≥ min and max = * represents an undefined number of amino acids.

Note that our representation based on graphs is a straightforward and intuitive method to describe the two-dimensional structure of a protein–ligand pattern. Additionally, the model has the potential to be expanded in order to accommodate other forms of structural patterns.

### GSP4PDB

GSP4PDB is a web application that allows users to create, search and analyze structural patterns of protein–ligand interactions. It is built on the graph-based representation method described in ‘Graph-based structural pattern’ section.

GSP4PDB consists of three main components: (i) GSP4PDB-parser, a Java tool that can be utilized to extract and preprocess data acquired from the PDB; (ii) a relational database (hosted in PostgreSQL) employed for storing and querying protein data; and (iii) a web application^2^, which offers a graphical interface for visualizing graph-based structural patterns and exploring the search results within the relational database.

**Figure 3.**
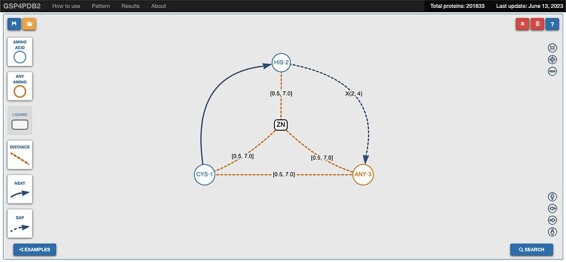
Navigation bar and design area of GSP4PDB. The navigation bar contains the components that can be used to draw the graph-based structural pattern in the design area. Alt Text: Navigation bar and design area of GSP4PDB.

**Figure 4.**
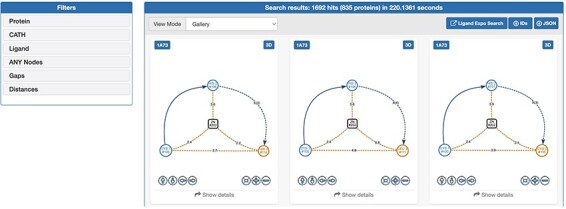
Output area of GSP4PDB. Each solution is a match of the graph-based structural pattern in a specific protein. Alt Text: Output area of GSP4PDB.

The web interface of GSP4PDB is divided into four main areas: the Navigation Bar, the Design area, the Output area and the About area (refer to Figures 3 and 4). The Navigation Bar is used for navigating through the elements of the interface and displays the number of proteins in the database. The Design area enables the user to create a GSP using a drag-and-drop interface with buttons corresponding to the types of nodes and edges allowed in a GSP. The Output area presents the search results of the GSP in the database and provides facets (filters) to further explore and analyze the results.

The usability of GSP4PDB is determined by its graphical user interface and how efficiently it can search for graph-structural patterns. In this paper, we will focus on evaluating the system’s efficiency. Specifically, we will examine the design and optimization of the relational database system that supports the graphical interface.

## Related work

In this section, we will provide a literature review on the storage methods employed for PDB data, alongside the database optimization techniques utilized. Moreover, we will incorporate details pertaining to optimization techniques employed in relational databases.

### Systems for managing PDB data

The literature that was reviewed includes studies that use relational databases to store data related to protein structures, including amino acids and ligands, among other data (2, 5, 11, 23). Alternatively, there are studies that store protein information in graph databases, using engines like Neo4J, for instance (1, 17).

Hooft *et al.* (18) describe a system that stores all PDB information in ASCII files. In the works of Aslam *et al.* (5) and Hooft *et al.* (18), the authors describe software applications to analyze the elements and structures of proteins in a more user-friendly way.

Many of the studies mentioned earlier failed to prioritize optimizing the database and evaluating the efficiency of the system. However, (2, 17) presented some improvements to the design of their systems. However, these optimizations are not focused on relational databases but rather on databases for graphs or XML.

### Optimization of relational databases

Sanders and Shin (32) discussed a general methodology for implementing optimization techniques in relational databases, with a specific focus on denormalization. Denormalization is described as a transitional phase between the logical and physical modeling of a database, emphasizing the application’s requirements. Furthermore, the article acknowledges certain drawbacks, including compromised data integrity and reduced user-friendliness, despite its advantages.

Hoffer *et al.* (16) explain a technique for optimizing a relational database. Their method involves denormalization, where multiple tables are combined into a single table to minimize the need for data collection in each query. However, there are some drawbacks to this approach, such as a higher probability of errors and data inconsistency. The authors also discuss another form of denormalization called partitioning, which represents a relation as multiple tables. Partitioning offers several advantages, including increased efficiency, local optimization, improved security, recovery and response time and load balancing. Nevertheless, there are also disadvantages to partitioning, including inconsistent information access time, modeling complexity and the need for additional space and time for updates. Additionally, the authors describe the use of indexes and provide recommendations on when it is advisable to use them.

Zhang *et al.* (39) introduce A-Store, an optimization algorithm for relational databases based on denormalization strategies to speed up the analytical processing of queries on in-memory data. The researchers compared the use of denormalization and analyzed the performance improvement it offers for different database engines. Nevertheless, using denormalization comes with some drawbacks, including the potential to introduce errors and cause data inconsistency.

Tsai and Kwee (36) conducted a study on various optimization methods for relational databases. The main goal of these approaches is to enhance data mining processes. Additionally, the study includes performance tests that utilize different optimization techniques for both data insertion and queries. The primary indexes employed are B-Tree and Hash, which can significantly enhance processing time for large data sets (around 500 000 records).

After reviewing the techniques, it is evident that denormalization and the use of indexes can enhance the performance of the database. This is mainly beneficial due to the substantial number of queries that are needed, and a normalized design would not be able to adequately meet this requirement. Moreover, implementing indexes would further enhance the performance, particularly in large databases with an abundance of queries. However, it is crucial to be cautious when using these techniques, especially in the case of denormalization. This is because it can result in duplicated information, thus requiring careful attention to ensure accurate updates and consistency of the information in the tables.

Finally, after reviewing the relevant studies, we have reached the conclusion that utilizing a relational database can result in favorable performance compared to other available options. However, it is worth mentioning that despite the extensive literature review, we did not come across any studies that specifically tackled the problem of searching for structural patterns within PDBs using a relational database. Our findings were limited to methods of storing PDBs in a database primarily intended for protein querying purposes.

## Relational database for proteins

GSP4PDB was designed to work with data acquired from the PDB (6, 30), which is the universal repository for structural data on proteins and nucleic acids. PDB is among the most extensively utilized resources in the fields of biology and biomedicine. As of July 2023, PDB comprises information on 206 987 macromolecular structures, including proteins, DNA and RNA.

The protein data are publicly available and can be downloaded from the PDB website^3^ in various formats. In this section, we will explain the procedure for extracting the data from the PDB files and how it is subsequently loaded into a relational database.

### Data extraction and pre-processing

The PDB dataset is organized as a set of data files, each one containing information about a single macromolecular structure (e.g. a protein, DNA or RNA). These data files are available in three different formats^4^: a textual-based format called PDB, a XML-based format called PDBML and a key-value representation called PDBx/mmCIF (which is the standard format since 2014). Some of the information included in a PDB file includes details about the authors, chains, amino acids, ligands, atomic coordinates and citation references.

We have developed gsp4pdb-parser, a command-line Java application that processes PDB files and exports the protein data either to comma-separated values files or to a relational database system (PostgreSQL). gsp4pdb-parser utilizes the biojava API^5^ to read the files. The current version of the parser can only process files that are encoded using the PDB format.

At first, the gsp4pdb-parser software examines a specified local directory chosen by the user to locate PDB files and creates a list of these files to be processed. This list is then filtered based on the proteins that are present in the PostgreSQL database. Optionally, the user also has the ability to define their own list of files to be processed. To ensure that the latest proteins from the primary PDB repository are included, we utilize rsync to maintain a local copy of the PDB dataset. Consequently, whenever gsp4pdb-parser is executed, the PostgreSQL database is updated with the most recent proteins that have been released in the primary PDB repository.

**Figure 5.**
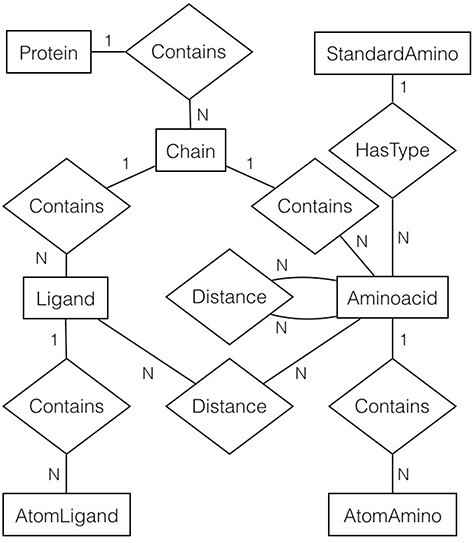
Entity-relationship diagram of the protein data used by GSP4PDB. It shows the entities, relationships and attributes identified and used to create the PostgreSQL database. Alt Text: Entity-relationship diagram of the GSP4PDB database.

For every file (or protein) in the filtered list, gsp4pdb-parser reads the file and generates a protein’s object model. The main classes of the model correspond to the entities and relationships described in the entity-relationship diagram shown in Figure 5. Note that the diagram is abbreviated as it does not contain the attributes for entities and relationships (which will be described later).

In general, a protein is made up of multiple chains. Each chain consists of ligands and amino acids, and both are composed of atoms. However, an amino acid is associated with a particular standard amino acid. There are two types of distance relationships: one connecting amino acids and the other connecting ligands with amino acids.

Even though a protein can have multiple chains, we only focus on processing the chain that holds more information. In other words, we prioritize the chain that has the highest number of amino acids and ligands. Note that a PDB file does not contain the atomic distances between amino acids and ligands. After conducting empirical tests, we have made the decision to pre-calculate these distances. This is aimed at enhancing the system’s performance since it involves intricate join operations [In the context of relational databases, a join operation is used to combine tuples (rows) from two different tables based on some common information. The Join is one of the most difficult operations to implement efficiently, as no predefined links between tables are required to exist (28).] for the relational database system.

The distance between two amino acids *A* and *A*^ʹ^ is calculated as the minimum distance between each pair of atoms $(a_i,a_j)$ such that $a_i \in A$ and $a_j \in A^{\prime}$ (i.e. we compute the distance between each pair of atoms of *A* and *A*^ʹ^). A similar approach is applied to determine the distance between a ligand *L* and an amino acid *A*. Distances greater than 7.0Å are not considered as we assume that there is no interaction between the atoms. Furthermore, the object model defines the class NextAminoAmino to store the adjacency relationship between two amino acids, i.e. the sort between each pair of amino acids in the chain.

After constructing the object model of the protein, the gsp4pdb-parser loads the data into the PostgreSQL database using a single bulk of SQL instructions. Next, we will explain the relational model that is utilized for the storage and management of protein data.

### Relational schema

**Figure 6.**
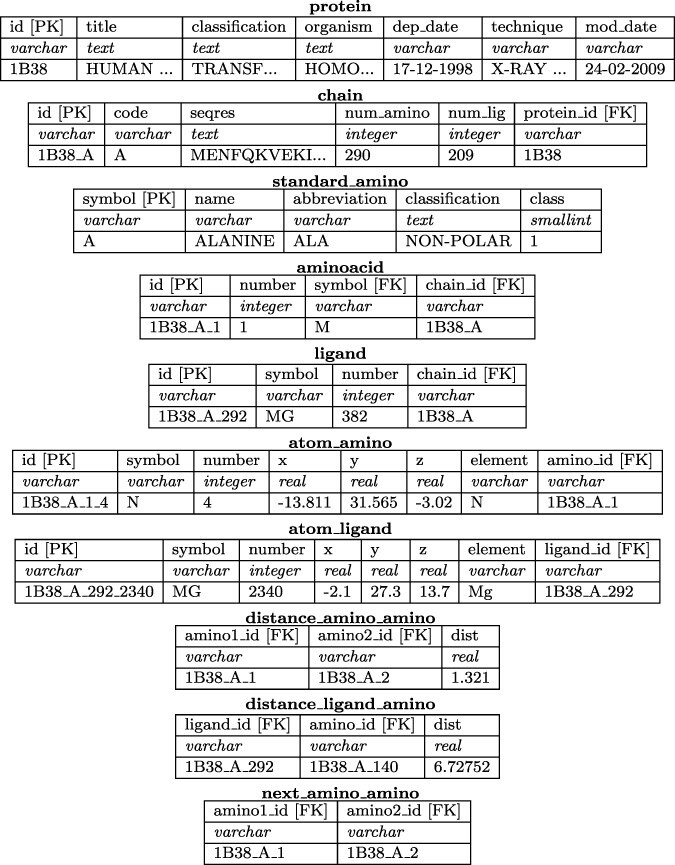
Structure (or relational schema) of the database for storing protein information. For each table, we show attributes (first row), data types (second row) and a sample data tuple (third row). Primary keys and foreign keys are marked as [PK] and [FK], respectively. Alt Text: Relational schema of the GSP4PDB database.

GSP4PDB uses the PostgreSQL database system (version 15) to store and retrieve protein data obtained from the PDB repository. We created a relational database schema based on the entity-relationship diagram presented in Figure 5. This schema comprises the tables listed in Table 1. Figure 6 displays the attributes (columns), data types and sample data for each table.

**Table 1.** Tables of the relational database used by GSP4PDB.

Table	Number of rows
protein	194 461
chain	144 881
standard_amino	21
aminoacid	45 365 304
ligand	558 092
atom_amino	380 064 372
atom_ligand	5 824 698
distance_amino_amino	413 783 764
distance_ligand_amino	9 138 739
next_amino_amino	45 220 493

The main components of the database are represented in the tables protein, chain, aminoacid, ligand, atom_amino and atom_ligand. The tables distance_amino_amino and distance_ligand_amino store information about the distances between amino acids and ligands. The table next_amino_amino maintains the sequential relationship between amino acids. Note that the information contained in these tables is not explicitly provided by PDB. Instead, it is computed during the pre-processing phase.

Note that the database has been normalized to reduce data redundancy and preserve data integrity. The attribute ID is used as the primary key in most tables, and each attribute that serves as a foreign key has a name that describes its purpose. Both primary keys and foreign keys are clearly labeled in Figure 6.

Note that the number of chains may not match the number of proteins, as certain PDB files contain nucleic acid information. In such cases, we keep fundamental information about the nucleic acid in the Protein table. The table standard_amino is the smallest one because it only contains information about the 20 standard amino acids and also includes an ‘undefined’ amino acid. The symbol for this amino acid is ‘U’, and its abbreviation is ‘UND’. The number of amino acids is greater than the number of ligands. The above statement explains why the largest tables are named atom_amino and distance_amino_amino.

The values used in the attributes named id are created (during pre-processing) to describe the provenance of the data. For instance, the tuple in table atom_amino having id equal to ‘1B38_A_1_4’ describes the atom number 4, which belongs to the amino acid number 1, of Chain ‘A’, in the protein ‘1B38’. Figure 6 also shows the attributes acting as foreign key, whose name indicates the referenced table.

### Translating graph-based structural patterns into SQL queries

Since we are utilizing a relational database for protein data storage, the conventional approach for searching and retrieving information from this type of database involves utilizing the SQL query language. Therefore, we have established a technique to convert a protein–ligand structural pattern into an SQL query expression.

In simple terms, the query translation method creates an SQL query expression for each subgraph pattern (node-edge-node) that appears in the main graph pattern. The final SQL query, which represents the main graph pattern, is composed of all these sub-expressions. These compositions can be easily accomplished by utilizing the natural join operator. However, despite its simplicity, this method is inefficient because it necessitates multiple join operations and comparisons for computing the final SQL query. Next, we will present a more in-depth explanation of the method and the issues it poses.

Consider the following subgraph patterns that can occur in a protein–ligand structural pattern:

1. Ligand Distance ⋯ Amino
2. Ligand ⋯ Distance ⋯ AnyAmino
3. AnyLigand ⋯ Distance ⋯ Amino
4. AnyLigand ⋯ Distance ⋯ AnyAmino
5. Amino—Distance — Amino
6. Amino—Distance — AnyAmino
7. AnyAmino—Distance — Amino
8. AnyAmino—Distance — AnyAmino
9. Amino—Next → Amino
10. Amino—Next → AnyAmino
11. AnyAmino—Next → Amino
12. AnyAmino—Next → AnyAmino
13. Amino—Gap → Amino
14. Amino—Gap → AnyAmino
15. AnyAmino—Gap → Amino
16. AnyAmino—Gap → AnyAmino

where the symbols ‘⋯’, ‘—’ and ‘→’ are used to resemble the three types of edges allowed by the graphical interface of GSP4PDB.

**Figure 7.**
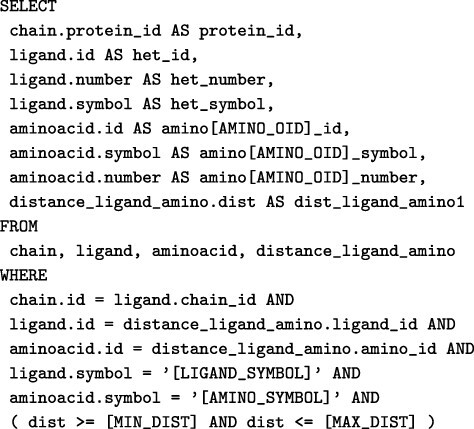
SQL query template for a subgraph pattern of the form Ligand ⋯ distance ⋯ Amino. The parameters of the template are represented using squared brackets (e.g. [AMINO_OID]). Alt Text: SQL query template for a subgraph pattern.

For each subgraph pattern, there is a SQL query template whose parameters are filled to generate a specific SQL query, also known as an ‘instance query’. For example, the SQL template associated with the subgraph pattern Ligand ⋯ Distance ⋯ Amino is shown in Figure 7.

Let *P* be a graph pattern formed by the subgraph patterns $P_1, \dots, P_n$. The SQL query *Q* obtained from *P* will have the structure:

SELECT *FROM (SELECT * FROM protein) AS SQINNER JOIN ( [Subquery_1] ) AS SQ1INNER JOIN…INNER JOIN ( [Subquery_2$ ) AS SQN

where Subquery_i denotes the SQL instance query corresponding to the subgraph pattern *P*_*i*_.

For instance, the graph pattern shown in Figure 3 is formed by the following subgraph patterns: ZN ⋯ Distance ⋯ CYS1, ZN ⋯ Distance ⋯ HIS2, ZN ⋯ Distance ⋯ ANY3, CYS1 – Next → HIS2, HIS2 – Gap → ANY3, CYS1 – Distance – ANY3. Therefore, the ultimate SQL query expression, which represents the entire graph pattern, will consist of SQL expressions formed for each subgraph pattern as described earlier.

### Evaluation of the normalized database

**Figure 8.**
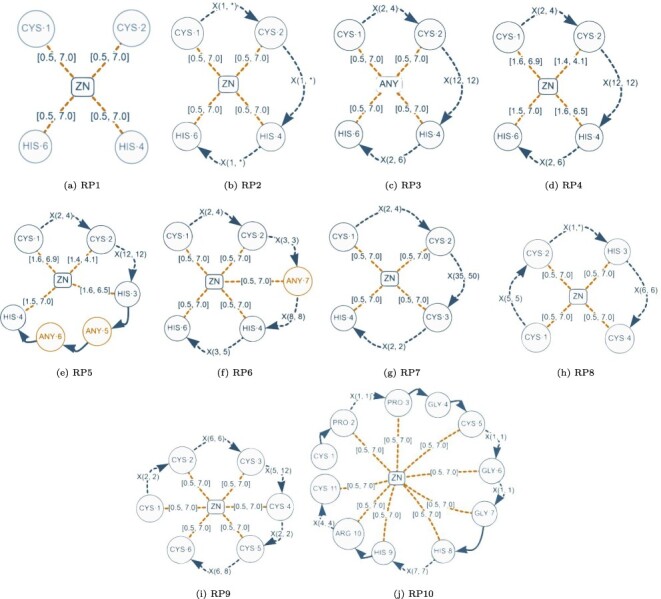
Graph-based structural patterns representing real protein–ligand structural patterns. Alt Text: Real graph-based structural patterns.

Although a normalized relational schema is suitable for storage, it is not efficient for responding to queries. To confirm this, we performed an empirical evaluation of the normalized database. The query performance evaluation was based on the protein–ligand structural patterns shown in Figure 8. These patterns are actual use-cases discovered in the literature and can be regarded as representative because they encompass all the elements identified in a protein–ligand structural pattern. The particular characteristics exhibited by each test pattern are displayed in Table 2.

**Table 2.** Characteristics of the graph-based structural patterns shown in Figure 8.

Element/pattern	RP1	RP2	RP3	RP4	RP5	RP6	RP7	RP8	RP9	RP10
Amino	4	4	4	4	4	4	4	4	6	11
AnyAmino	–	–	–	–	2	1	–	–	–	–
Ligand	1	1	–	1	1	1	1	1	1	1
AnyLigand	–	–	1	–	–	–	–	–	–	–
Next	–	–	–	–	3	–	–	–	–	5
Gap	–	3	3	3	2	4	3	3	5	5
DistLigandAmino	4	4	4	4	4	5	4	4	6	9
#Elements	9	12	12	12	16	15	12	12	18	31

This table shows the quantity of elements present in each pattern.

For every real pattern RPX, we have generated an SQL query QX using the query translation method that is described in the ‘Translating graph-based structural patterns into SQL queries’ section. In the initial experiment, we performed all the SQL queries on the normalized database without implementing any kind of optimization. Surprisingly, the database was unable to respond to the queries. In the second experiment, we added B-tree indexes in the following attributes and tables: (i) attribute symbol in table aminoacid; (ii) attribute symbol in table ligand; (iii) attributes amino1_id and amino2_id in table distance_amino_amino; (iv) attributes ligand_id and amino_id in table distance_ligand_amino; and (v) attributes amino1_id and amino2_id in table next_amino_amino. Indexes (i) and (ii) can improve the search of amino acids and ligands by using the attribute symbol. The rest of indexes were included to improve the evaluation of joins.

The running times of the normalized database with the aforementioned basic indexing schema are shown in Table 3. Even though we received responses for certain queries, the amount of time taken for execution was not satisfactory. Next, we will discuss a proposal aimed at enhancing the database through the implementation of denormalization and indexing.

**Table 3.** Runtimes of the normalized database with indexes.

Query	Q1	Q2	Q3	Q4	Q5
Runtime	–	–	–	–	33.905
Query	Q6	Q7	Q8	Q9	Q10
Runtime	49.507	–	8.027	15.545	206.046

For each SQL test query (e.g. Q1), we indicate its runtime (in milliseconds) or a line (–) in the case of time-out (10 min).

## Database optimization

At this point, we have explained how to use a relational database to store protein data, and we have also proved that this database is not sufficient for effectively searching for protein–ligand structural patterns. In this section, we will discuss two methods that can enhance the performance of the database: denormalization and indexing.

### Denormalization

Denormalization optimizes database performance by including redundant data, which minimizes the reliance on join operations (25). This approach streamlines data retrieval by consolidating the information into fewer tables, thereby simplifying query execution (35).

There are several types of denormalization, including pre-joined tables, report tables, mirror tables, split tables, combined tables, redundant data, repeating groups, derivable data and speed tables (32). While denormalization has been proven to be useful in numerous cases, the authors suggested that it should be cautiously implemented based on how the data will be queried.

In this case, we implemented data redundancy to minimize the need for joining and merging tables. Specifically, we duplicate the attributes protein.id, aminoacid.symbol, aminoacid.class, aminoacid.number, ligand.symbol and ligand.number in tables distance_amino_amino, distance_ligand_amino and next_amino_amino. This creates the denormalized tables below, where the repeated attributes are indicated with +:

distance_amino_amino (protein_id+,amino1_id, amino1_symbol+, amino1_class+,amino1_number+, amino2_id, amino2_symbol+,amino2_class+, amino2_number+, dist)

distance_ligand_amino (protein_id+,ligand_id, ligand_symbol+, ligand_number+,amino_id, amino_symbol+, amino_class+,amino_number+, dist)

next_amino_amino (protein_id+, amino1_id,amino1_symbol+, amino1_class+,amino1_number+, amino2_id, amino2_symbol+,amino2_class+, amino2_number+)

The schema aforementioned offers the following advantages: the attribute protein_id avoids the join with table protein; the attributes with postfix _symbol, _class and _number avoid the joins with tables aminoacid and ligand. Thus, the three tables mentioned earlier contain the necessary data to search for structural patterns of any protein–ligand type.

To assess the influence of denormalization, we examined the test graph patterns described in ‘Evaluation of the normalized database’ section. Note that the corresponding SQL queries require fewer joins and fewer tables than the ones created for the normalized database. In this situation, the database was able to respond to all the SQL test queries, although the execution time remained high when handling complex patterns. Thus, through experimentation, we confirmed that by employing denormalization, we can partially enhance the database. However, this approach must be supplemented with other optimization techniques, which we will soon discuss in detail.

### Indexing

An index is a data structure plus a method of arranging the data tuples in the table being indexed (25). An index helps to find and retrieve specific tuples much faster than without an index. B+-trees and Hash structures are the two types of indexes that are most commonly used in practice.

Based on the literature (22, 33) and the technical documentation of PostgreSQL^6^, we decided to introduce B+tree indexes in two cases: (i) in attributes working as foreign key, so PostgreSQL can use an efficient join implementation when necessary and (ii) in attributes used for filtering node-edge-node patterns. The specific indexed attributes are the following:

- Attributes amino1_id, amino1_symbol, amino2_id and amino2_symbol in table distance_amino_amino;
- Attributes amino_id, amino_symbol, het_id and het_symbol in table distance_ligand_amino; and
- Attributes amino1_id, amino1_symbol, amino2_id and amino2_symbol in table next_amino_amino.

It is worth noting that although indexes are beneficial for enhancing query speed, it is also widely recognized that they can affect database manipulation such as insert, update and delete operations due to the need for index reconstruction. However, this is not a concern in our case since protein data remain constant over time. Next we will present the experimental evaluation of the denormalized and indexed database.

## Experimental evaluation

This section presents an empirical evaluation of the optimized database described in ‘Database optimization’ section. In particular, we assess the performance of the database that has been denormalized and indexed by conducting tests using both real and artificial protein-ligand structural patterns on two separate machines. Following that, we outline the methodology of the evaluation, present the experimental results and provide the corresponding analysis.

### Methodology

Our empirical evaluation includes multiple experiments, where each experiment involves executing a SQL query on a database hosted on a specific computer machine. In this context, each experiment represents a specific combination of three variables: the database, computer machine and the query.

#### Databases

We are assessing two databases: DB1, which is the denormalized database, and DB2, the denormalized and indexed database. Both databases have the same relational schema. The tables, number of rows and storage size are summarized in Table 4. Please be informed that we have not taken into account the normalized database in our analysis as it was incapable of responding to the test queries.

**Table 4.** Tables of the optimized relational database.

Table	Row count	Size in hard disk
protein	194,161	42.21 MB
standard_amino	21	32 KB
distance_amino_amino	413,784,540	46.53GB
distance_ligand_amino	9,139,045	1.23 GB
next_amino_amino	45,220,448	7.79 GB

#### Computer machines

We utilized two machines, the computational specifications of which are specified in Table 5. When it comes to processing power, Machine 1 (M1) can be regarded as a standard computer as it possesses a hardware configuration suitable for general purposes. On the other hand, Machine 2 (M2) is a computer optimized for memory usage. It is capable of handling substantial datasets in the main memory.

**Table 5.** Computer machines used in the experimental evaluation.

	Machine 1 (M1)	Machine 2 (M2)
CPU	Intel Xeon E5-2609v3	Intel Xeon E5-2620
RAM	8 GB DDR3	96 GB DDR3
HDD	500 GB	2 TB
S.O.	Ubuntu Server 22.04	
DB	PostgreSQL 14	

#### Queries

Our empirical evaluation includes both real (‘Evaluation of real graph-based structural patterns’ section) and artificial (‘Evaluation of artificial graph-based structural patterns’ section) protein–ligand structural patterns. For each pattern, we have created a SQL query based on the query translation method described in ‘Translating graph-based structural patterns into SQL queries’ section.

#### Query test

A query test is a procedure that involves running a SQL query on a computer machine *M*, which hosts a database *D*. The terminal-based interface of PostgreSQL is utilized to perform each query test. The fastest running time achieved after running the query three times will be recorded as the result, with a maximum time-out limit of 10 min. The effectiveness of a database’s query performance will be indicated by the running times of the query tests performed on it.

### Evaluation of real graph-based structural patterns

This section describes our evaluation of the optimized databases by using the real protein–ligand structural patterns shown in the ‘Relational database for proteins’ section. Remember that these patterns were used to test the normalized database described in the ‘Relational database for proteins’ section, revealing its poor performance. Table 6 shows the query runtimes obtained for DB1 (denormalized database) and DB2 (denormalized and indexed database) running on machine M1. Table 7 shows the results obtained with machine M2.

**Table 6.** Empirical evaluation of real graph patterns on machine M1.

Pattern	Number of hits	DB1	DB2	% Imp
RP1	84 528	8.782	10.570	-20.35
RP2	2906	5.350	2.231	58.29
RP3	793	5.180	1.468	71.66
RP4	755	5.189	0.893	82.79
RP5	8	14.778	1.422	90.37
RP6	61	6.885	1.248	81.87
RP7	39	5.188	3.490	32.72
RP8	6	5.125	0.746	85.44
RP9	32	8.347	0.991	88.12
RP10	1	28.493	1.602	94.37

For each pattern, we show the number of solutions (number of hits), the runtime on database DB1 (in seconds), the runtime on database DB2 (in seconds) and the percentage of improvement (% Imp).

**Table 7.** Empirical evaluation of real graph patterns on Machine M2.

Pattern	Number of hits	DB1	DB2	% Imp
RP1	84 528	7.639	7.556	1.08
RP2	2906	5.025	2.037	59.46
RP3	793	5.087	1.422	72.04
RP4	755	5.051	0.903	82.12
RP5	8	13.671	1.276	90.66
RP6	61	6.571	1.134	82.74
RP7	39	4.799	3.075	35.92
RP8	6	4.958	0.697	85.94
RP9	32	7.995	0.922	88.46
RP10	1	25.944	1.056	95.92

For each pattern, we show the number of solutions (number of hits), the runtime on database DB1 (in seconds), the runtime on database DB2 (in seconds) and the percentage of improvement (% Imp).

When comparing the runtimes of the normalized database (Table 3) and the optimized database DB1 (Table 6), one notable difference is that DB1 provided a response for every query, while the normalized database failed to answer five queries. We can observe that DB1 reduced the runtime for the queries that are supported by the normalized database.

If we compare the performance of DB1 and DB2, both running on machine M1 (Table 6), we can see that DB2 reduces the query runtime in most cases, with an improvement reaching up to 94 percent for pattern RP10. A similar trend can be seen for the patterns executed on machine M2 (Table 7).

**Figure 9.**
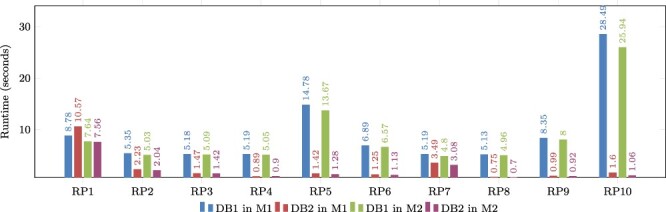
Comparison of runtimes obtained for real graph-based structural patterns. Note that RPX = real graph pattern X, DB1 = Denormalized database, DB2 = Denormalized and indexed database, M1 = Machine 1 (8-GB RAM) and M2 = Machine 2 (92-GB RAM). Alt Text: The runtimes for real graph-based structural patterns.

Figure 9 shows a graphical comparison of the runtimes obtained during the evaluation of real graph patterns. It is evident that DB2 consistently has lower runtimes compared to DB1. The only exception is in the evaluation of RP1 on Machine 1, where DB1 performs better, but this is not the case for Machine M2. This suggests that RP1 requires a sufficient amount of main memory in order to be executed efficiently. Notably, patterns RP5 and RP10 have the longest runtimes, but their performance can be enhanced by utilizing indexes. This also applies to the majority of other patterns.

It is important to note that the experiments conducted on both machines revealed a common factor: the response time of each pattern depends on two factors. First, it is conditioned by the number of results retrieved from the database when queried. Second, it is dependent on the complexity level of the pattern. It is observed that pattern RP1 is the least complex in terms of the relationships it uses. However, because it is a more commonly found pattern in proteins, it returns a higher number of results due to the abundance of matching data. Even though RP1 takes longer to process due to the larger result set, other patterns that return fewer results are not significantly affected by response time when indexing is used as an optimization, as mentioned before. Conversely, more complex patterns like RP5 and RP10 return fewer results, but their performance is influenced by different optimizations. The use of indexes in these cases improved their resolution time by more than 90 percent, regardless of the machine being used.

### Evaluation of artificial graph-based structural patterns

This section presents our evaluation of the optimized databases using artificial protein–ligand structural patterns. The aim was to create a set of patterns that would sufficiently challenge the databases. We first explain the process used to create the artificial patterns, followed by the presentation of the experimental results.

The artificial graph patterns were created by following the following process. First, we execute a query on the table to retrieve three ligands in the database with the highest prevalence in proteins. Next, for each chosen ligand, we retrieve the associated amino acids, determine their frequency and select the two amino acids with the highest frequency.

Table 8 displays the ligands that were chosen and their corresponding amino acids. The selected ligands are GLYCEROL (GOL), ETHANEDIOL (EDO) and SULFATE ION (SO4). The selected amino acids are LEUCINE (LEU), ARGININE (ARG), GLUTAMIC ACID (GLU) and LYSINE (LYS). Note that these ligands and amino acids can be utilized for constructing patterns that have a high likelihood of generating a substantial quantity of outcomes. However, we also consider the zinc ligand (ZN) and the CYS and HIS amino acids, as they were used in the real graph patterns evaluated in the ‘Evaluation of real graph-based structural patterns’ section.

**Table 8.** Ligands with the highest occurrence (number of hits) in the database and amino acids related to the highest frequency.

Ligand	Number of hits	Amino acid 1	Amino acid 2
GOL	578 761	LEU	ARG
EDO	570 850	LEU	GLU
SO4	539 010	ARG	LYS

**Figure 10.**
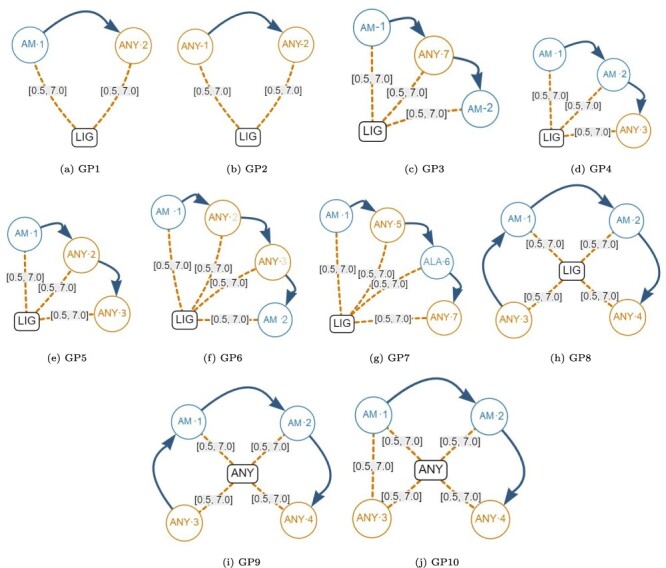
Generic graph-based structural patterns. These were used to create many artificial graph patterns. Alt Text: Generic graph-based structural patterns.

Next, we created the set of generic graph-based structural patterns shown in Figure 10. Each generic pattern contains a subset of all the possible elements allowed in a graph-based structural pattern. For example, the generic graph pattern GP1 contains a Ligand Node (LIG), an Amino node (AM-1), an AnyAmino node (ANY-2), a Next edge and two Distance edges (both with a distance range between 0.5Å and 7.0Å). The elements that are covered by each individual generic graph pattern are displayed in Table 9. Note that Gap edges and fixed distances were not included in the generic graph patterns because they do not impose any additional stress on the database.

**Table 9.** Coverage of the generic artificial graph patterns shown in Figure 10.

Element/pattern	GP1	GP2	GP3	GP4	GP5	GP6	GP7	GP8	GP9	GP10
Amino	1	–	2	2	1	2	2	2	2	2
AnyAmino	1	2	1	1	2	2	2	2	2	2
Ligand	1	1	1	1	1	1	1	1	–	–
AnyLigand	–	–	–	–	–	–	–	–	1	1
Next	1	1	2	2	2	3	3	3	3	2
Distance	2	2	3	3	3	4	4	4	4	5
#Elements	6	6	9	9	9	12	12	12	12	12

To create a specific artificial graph pattern for query testing, we started with a generic graph pattern and replaced the elements with the most commonly found ligands and amino acids that were identified at the beginning of the process. As a result, we generated 10 distinct artificial graph patterns, and their respective elements can be seen in Table 10.

**Table 10.** Specific artificial graph patterns.

Artificial pattern	Ligand (LIG)	Aminoacid 1 (AM-1)	Aminoacid 2 (AM-2)
AP1	ZN	CYS	
	EDO	LEU	
	GOL	LEU	
	SO4	ARG	
AP2	ZN		
	EDO		
	GOL		
	SO4		
AP3	ZN	CYS	CYS
	EDO	LEU	GLU
	GOL	ARG	LEU
	SO4	LYS	ARG
AP4	ZN	CYS	CYS
	EDO	LEU	
	GOL	LEU	
	SO4	ARG	
AP5	ZN	CYS	
	EDO	LEU	
	GOL	LEU	
	SO4	ARG	
AP6	ZN	CYS	HIS
	EDO	LEU	GLU
	GOL	LEU	ARG
	SO4	LYS	ARG
AP7	ZN	CYS	
	EDO	LEU	
	GOL	LEU	
	SO4	LYS	
AP8	ZN	CYS	HIS
	EDO	GLU	LEU
	GOL	LEU	ARG
	SO4	LYS	ARG
AP9	ZN	CYS	HIS
	EDO	GLU	LEU
	GOL	LEU	ARG
	SO4	LYS	ARG
AP10	ZN	CYS	HIS
	EDO	GLU	LEU
	GOL	ARG	LEU
	SO4	ARG	LYS

The empirical results derived from evaluating the artificial graph patterns on databases DB1 and DB2, while running on machine M2, are presented in Table 11. We can observe a significant improvement in the runtimes of patterns AP1, AP3-AP8 and AP10 when denormalization was utilized in conjunction with the implementation of indexes (DB2). Specifically, artificial patterns AP3, AP4, AP5, AP7 and AP10 demonstrate that the utilization of indexes resulted in significant improvements. These improvements include timely response within the defined response time and considerably reduced durations for the majority of cases. The problem of pattern AP10 is challenging to solve because of the range of its elements, where the distance relationship holds importance. This is different from cases where denormalization was the only approach used, but it failed to provide results within the designated time frame.

**Table 11.** Empirical evaluation of artificial graph patterns on Machine 2. For each pattern, we show the evaluated ligand, the number of solutions (# Hits), the runtime on database DB1 (in seconds), the runtime on database DB2 (in seconds), and the percentage of improvement (% Imp). AEach pattern was executed three times with a maximum execution time of six minutes (marked as >360).

Artificial pattern	Ligand	# Hits	DB1	DB2	% Imp
AP1	ZN	44,865	4.905	2.563	47.75%
	EDO	26,848	4.831	1.623	66.40%
	GOL	25,084	4.673	1.8	61.48%
	SO4	24,517	4.516	1.682	62.75%
AP2	ZN	244,187	15.067	61.029	-305.05%
	EDO	338,033	18.759	79.079	-321.55%
	GOL	341,293	18.645	87.771	-370.75%
	SO4	299,455	17.14	63.596	-271.04%
AP3	ZN	1,974	>360	0.845	
	EDO	1,598	>360	0.9	
	GOL	1,020	>360	1.2	
	SO4	759	>360	1.145	
AP4	ZN	1,169	>360	2.42	
	EDO	1,325	>360	2.357	
	GOL	1,150	>360	2.235	
	SO4	1,029	6.618	2.061	68.86%
AP5	ZN	34,262	>360	2.989	
	EDO	18,616	10.656	2.26	78.79%
	GOL	17,717	10.432	2.406	76.94%
	SO4	13,415	>360	2.323	
AP6	ZN	1,567	20.125	1.99	90.11%
	EDO	709	19.175	1.486	92.25%
	GOL	660	20.041	1.673	91.65%
	SO4	588	16.589	1.773	89.31%
AP7	ZN	1,479	>360	1.124	
	EDO	889	>360	1.393	
	GOL	1,005	>360	1.485	
	SO4	470	>360	1.136	
AP8	ZN	1,034	>360	7.982	
	EDO	777	>360	1.344	
	GOL	650	27.178	4.173	84.65%
	SO4	503	>360	3.651	
AP9	ZN	2,917	16.238	3.176	80.44%
	EDO	8,589	45.868	>360	
	GOL	8,530	50.487	>360	
	SO4	4,375	31.928	>360	
AP10	ZN	14,866	>360	90.177	
	EDO	8,837	>360	311.341	
	GOL	3,138	>360	146.916	
	SO4	5,660	>360	303.239	

The artificial patterns AP2 and AP9 display the most favorable behavior in the database DB1. This occurs because a significant number of comparisons are necessary when employing the ligand of type ANY. This is the case with pattern AP9 or when selecting all amino acids as ANY, as seen with pattern AP2. The use of indexes becomes inefficient due to the large number of comparisons that need to be made. Additionally, the clustering methods result in a longer query processing time, which, in some cases, leads to no responses being generated. This is the case of pattern AP9, for three out of the four cases, in which the presence of a higher number of amino acids linked to ligands leads to a decrease in the speed of query processing when indexes are employed, contrary to the anticipated improvement. It is important to recognize that although some patterns had worse performance when indexes were used, this does not imply that the use of indexes is a bad optimization. In general, using indexes led to improved performance for most of the tested queries.

## Conclusions and future work

This article demonstrates that employing denormalization and indexing enhances the query performance of a relational database designed for storing protein data and querying graph-based structural patterns. Compared to the original normalized database, the denormalized database was capable of computing all the actual and artificial patterns utilized in our experimental evaluation. Additionally, the implementation of indexes also enhanced the database’s performance, although this was only observed in certain cases. By combining both techniques, we were able to get 90% of improvement for some real patterns.

Because our database is continuously expanding due to the delivery of new proteins every week, it is necessary to explore alternative optimization methods. Currently, we are in the process of testing the rewriting of nested queries.

We noticed that the time it takes to execute a SQL query (created for a specific graph pattern) is affected by the order of the related subqueries (each one corresponding to a sub-pattern of node-edge-node). For instance, Table 12 displays the execution times achieved for the identical query, albeit with different arrangements of the subqueries taken into account. Note that each of the runtimes varies. The first combination displayed signifies the shortest runtime. Therefore, we aim to establish a method for selecting the optimal combination and, consequently, diminish the query’s runtime.

**Table 12.** Runtimes for the same SQL query (i.e. a protein-ligand pattern), but changing the order of the subqueries.

	Order	Runtime (sec.)
1	Ligand ⋯ Distance ⋯ Amino Amino — Next → AnyAmino Ligand ⋯ Distance ⋯ AnyAmino	2.206
2	Amino — Next → AnyAmino Ligand ⋯ Distance ⋯ AnyAmino Ligand ⋯ Distance ⋯ Amino	2.361
3	Ligand ⋯ Distance ⋯ Amino Ligand ⋯ Distance ⋯ AnyAmino Amino — Next → AnyAmino	2.348
4	Amino — Next → AnyAmino Ligand ⋯ Distance ⋯ Amino Ligand ⋯ Distance ⋯ AnyAmino	2.359

## Footnotes

1
https://prosite.expasy.org/prosuser.html#conv_pa

2
http://dbg.utalca.cl/gsp4pdb/gsp4pdb2/

3
https://www.rcsb.org

4
https://www.rcsb.org/pdb/static.do?p=file_formats/index.jsp

5
http://biojava.org/

6
https://www.postgresql.org/docs/current/indexes.html

## References

[1] Dhifli Abdoulaye W. (2015) PGR: a novel graph repository of protein 3D-structures. *J. Data Mining in Genomics & Proteomics*, 6, 1–4.

[2] Anders G. and Nicola M. (2011). *Managing the Protein Data Bank with DB2 pureXML* IBM developerWorks, Technical Library.

[3] Angles R. and Arenas M. (2018) A graph-based approach for querying protein-ligand structural patterns. In: *Lecture Notes in Bioinformatics*, 10813, Springer, Cham, pp. 235–244.

[4] Angles R. , Arenas-Salinas M., García R. et al (2020) GSP4PDB: A web tool to visualize, search and explore protein-ligand structural patterns. *BMC Bioinform.*, 21, 1–15. 10.1186/s12859-020-3352-x PMC7068854 32164553

[5] Aslam N. , Nadeem A. and Ellahi Babar M. et al (2016) RPDB: A relational databank of protein structures. *Pak. J. Agric. Sci.*, 53, 129–134.

[6] Berman H.M. , Westbrook J. and Feng Z. et al (2000) The Protein Data Bank. *Nucleic Acids Res.*, 28, 235–242. 10592235 10.1093/nar/28.1.235 PMC102472

[7] Bittrich S. , Burley S.K. and Rose A.S. (2020) Real-time structural motif searching in proteins using an inverted index strategy. *PLoS Comput. Biol.*, 16, 1–17. 10.1371/journal.pcbi.1008502 PMC7746303 33284792

[8] Branden C. and Tooze J. (1998) *Introduction to Protein Structure*, 2nd edn., Garland Science, USA.

[9] Cassandri M. , Smirnov A., Novelli F. et al (2017) Zinc-finger proteins in health and disease. *Cell Death Discov.*, 3, 17071. 10.1038/cddiscovery.2017.71 PMC5683310 29152378

[10] Cia G. , Marc Kwasigroch J., Stamatopoulos B., Rooman M. and Pucci F. (2023) pyScoMotif: Discovery of similar 3D structural motifs across proteins. *Bioinformatics Advances* 3, 1. 10.1093/bioadv/vbad158 PMC10640396 38023327

[11] Davis F.P. and Sali A. (2005) PIBASE: a comprehensive database of structurally defined protein interfaces. *Bioinformatics*, 21, 1901–1907. 15657096 10.1093/bioinformatics/bti277

[12] Diedrich K. , Graef J. and Schöning-Stierand K., et al (2020) GeoMine: interactive pattern mining of protein-ligand interfaces in the Protein Data Bank. *Bioinformatics*, 37, 424–425. 10.1093/bioinformatics/btaa693 32735322

[13] Ehrt C. , Brinkjost T. and Koch O. (2016) Impact of binding site comparisons on medicinal chemistry and rational molecular design. *J. Med. Chem.*, 59, 4121–4151. 27046190 10.1021/acs.jmedchem.6b00078

[14] Galperin M.Y. , Rigden D.J. and Fernández-Suárez X.M. (2015) The 2015 Nucleic Acids Res. Database Issue and Molecular Biology Database Collection. *Nucleic Acids Res.*, 43, D1. 10.1093/nar/gku1241 PMC4383995 25593347

[15] Grinter S.Z. and Zou X. (2014) Challenges, applications, and recent advances of protein-ligand docking in structure-based drug design. *Molecules*, 19, 10150–10176. 25019558 10.3390/molecules190710150 PMC6270832

[16] Hoffer J.A. , Ramesh V. and Topi H. (2016) *Modern Database Management*, 12th edn., London , England: Pearson Education Limited.

[17] Hoksza D. and Jelinek J. (2015) Using Neo4j for mining protein graphs: a case study. In: *26th International Workshop on Database and Expert Systems Applications (DEXA)*. IEEE: Valencia, Spain. 230–234.

[18] Hooft R.W.W. , Sander C. and Scharf M., et al (1996) The PDBFINDER database: a summary of PDB, DSSP and HSSP information with added value. *Bioinformatics*, 12, 525–529. 10.1093/bioinformatics/12.6.525 9021272

[19] Iuchi S. (2001) Three classes of C2H2 zinc finger proteins. *Cellular and Molecular Life sciences*, 58, 625—635. 11361095 10.1007/PL00000885 PMC11146492

[20] Klug A. (2010) The discovery of zinc fingers and their applications in gene regulation and genome manipulation. *Annu. Rev. Biochem.*, 79, 213–231. 20192761 10.1146/annurev-biochem-010909-095056

[21] Konc J. and Janežič D. (2014) Binding site comparison for function prediction and pharmaceutical discovery. *Curr. Opin. Struct. Biol.*, 25, 34–39. 24878342 10.1016/j.sbi.2013.11.012

[22] Kumar V.N.A. (2021) *PostgreSQL 13 Cookbook*, Birmingham, UK: Packt Publishing.

[23] Lee W. , Yu W. and Kim S. et al (2012) PACSY, a relational database management system for protein structure and chemical shift analysis. *J. Biomol. NMR*, 54, 169–179. 22903636 10.1007/s10858-012-9660-3 PMC3542970

[24] Lesk A. (2010) *Introduction to Protein Science: Architecture, Function, and Genomics*, 2nd edn. Oxford University Press, UK.

[25] Liu L. and Tamer Ozsu. M. (2009) *Encyclopedia of Database Systems*, New York, USA: Springer.

[26] Mavromoustakos T. , Durdagi S. and Koukoulitsa C. et al (2011) Strategies in the Rational Drug Design. *Current Medicinal Chemistry*, 18, 2517–2530. 21568895 10.2174/092986711795933731

[27] Meysman P. , Zhou C., Cule B. et al (2015) Mining the entire Protein DataBank for frequent spatially cohesive amino acid patterns. *BioData Min.*, 8, 4. 10.1186/s13040-015-0038-4 PMC4318390 25657820

[28] Mishra P. and Eich M.H. (1992) Join processing in relational databases. *ACM Comput. Surv.*, 24, 63–113.

[29] Berman H. M. , Westbrook J, Feng Z, et al (2000) The Protein Data Bank. *Nucleic Acids Res.*, 28, 1. 10.1093/nar/28.1.235 PMC102472 10592235

[30] Rose P.W. , Prlić A. and Altunkaya A. et al (2017) The RCSB protein data bank: integrative view of protein, gene and 3D structural information. *Nucleic Acids Res.*, 45, D271–D281. 27794042 10.1093/nar/gkw1000 PMC5210513

[31] Samish I. , MacDermaid C.M. and Manuel Perez-Aguilar J., et al (2011) Theoretical and computational protein design. *Annu. Rev. Phys. Chem.*, 62, 129–149. 21128762 10.1146/annurev-physchem-032210-103509

[32] Sanders G. and Shin S. (2001) Denormalization effects on performance of rdbms. In: *Proceedings of the 34th Annual Hawaii International Conference on System Sciences (HICSS)*. IEEE Computer Society, USA.

[33] Sasha D. and Bonnet P. (2002) *Database Tuning: Principles, Experiments and Troubleshooting Techniques*, Morgan Kaufmann, San Francisco, CA, USA.

[34] Schierz A.C. , Soldatova L.N. and King R.D. (2007) Overhauling the PDB. *Nat. Biotechnol.*, 25, 437–442. 17420753 10.1038/nbt0407-437

[35] Shin S.K. and Lawrence Sanders. G. (2006) Denormalization strategies for data retrieval from data warehouses. *Decis. Support Syst.*, 42, 267–282.

[36] Tsai F.S. and Kwee A.T. (2011) Database optimization for novelty mining of business blogs. *Expert Syst. Appl.*, 38, 11040–11047.

[37] Williams M.A. (2013) *Protein–Ligand Interactions: Fundamentals*, Humana Press, New York, NY, pp. 3–34. 10.1007/978-1-62703-398-5_1 23729247

[38] Mark A.W. (2013) Protein-ligand interactions: Fundamentals. *Methods mol. biol.*, 1008, 3–34. 23729247 10.1007/978-1-62703-398-5_1

[39] Zhang Y. , Zhou X. and Zhang Y. et al (2016) Virtual Denormalization via Array Index Reference for Main Memory OLAP. *IEEE Trans. Knowl. Data Eng.*, 28, 1061–1074.

